# Lymphocyte respiration in children with Trisomy 21

**DOI:** 10.1186/1471-2431-12-193

**Published:** 2012-12-18

**Authors:** Elhadi H Aburawi, Abdul-Kader Souid

**Affiliations:** 1 Department of Pediatrics, UAE University, P.O. Box 17666, Al Ain, United Arab Emirates

**Keywords:** Oxygen, Respiration, Mitochondria, Trisomy 21, Hypothyroidism

## Abstract

**Background:**

This study measured lymphocyte mitochondrial O_2_ consumption (cellular respiration) in children with trisomy 21.

**Methods:**

Peripheral blood mononuclear cells were isolated from whole blood of trisomy 21 and control children and these cells were immediately used to measure cellular respiration rate. [O_2_] was determined as a function of time from the phosphorescence decay rates (1/τ) of Pd (II)-*meso*-tetra-(4-sulfonatophenyl)-tetrabenzoporphyrin. In sealed vials containing lymphocytes and glucose as a respiratory substrate, [O_2_] declined linearly with time, confirming the zero-order kinetics of O_2_ conversion to H_2_O by cytochrome oxidase. The rate of respiration (*k*, in μM O_2_ min^-1^), thus, was the negative of the slope of [O_2_] *vs.* time. Cyanide inhibited O_2_ consumption, confirming that oxidation occurred in the mitochondrial respiratory chain.

**Results:**

For control children (age = 8.8 ± 5.6 years, n = 26), the mean (± SD) value of *k*_*c*_ (in μM O_2_ per min per 10^7^ cells) was 1.36 ± 0.79 (coefficient of variation, Cv = 58%; median = 1.17; range = 0.60 to 3.12; -2SD = 0.61). For children with trisomy 21 (age = 7.2 ± 4.6 years, n = 26), the values of *k*_*c*_ were 0.82 ± 0.62 (Cv = 76%; median = 0.60; range = 0.20 to 2.80), *p*<0.001. Similar results (*p*<0.000) were obtained after excluding the five trisomy 21 children with elevated serum TSH (values >6.1 mU/L). Fourteen of 26 (54%) children with trisomy 21 had *k*_*c*_ values of 0.20 to 0.60 (i.e., <−2SD). The values of *k*_*c*_ positively correlated with body-mass index (BMI, *R* >0.302), serum creatinine (*R* >0.507), blood urea nitrogen (BUN, *R* >0.535) and albumin (*R* >0.446).

**Conclusions:**

Children with trisomy 21 in this study have reduced lymphocyte bioenergetics. The clinical importance of this finding requires further studies.

## Background

Trisomy 21 is the most common chromosomal anomaly worldwide, affecting about 1 in 700 newborns [1]. These individuals typically have low resting metabolic rates [2] and are particularly susceptible to infections [3] and hypothyroidism [4]. Moreover, defects in the inner mitochondrial membrane potential [5] and mitochondrial respiratory chain enzymes are documented in these patients [6,7]. Mitochondrial disturbances, increased oxidative stress and apoptosis have been described in the neurons, predisposing to precocious Alzheimer’s disease [8]. Alterations in metabolic enzymes (e.g., monoamine oxidase-B, cytochrome oxidase, isocitrate dehydrogenase and glutamate dehydrogenase) have been also linked to impaired energy metabolism in trisomy 21 children [9]. Calcium levels are lower than in control children, which may alter cellular signaling [10].

Increased congenital heart disease and other major anomalies are exceptionally frequent in children with trisomy 21. It is not known whether these defects are linked to the biological impairments described above.

The use of the phosphorescence oxygen analyzer to measure lymphocyte respiration was recently reported. Lymphocytes were shown to be suitable for screening of certain mitochondrial disorders [11]. These methodologies were used to measure lymphocyte respiration rates in children with trisomy 21 and compare them with rates in children without this disorder.

## Methods

### Reagents and solutions

Pd (II) complex of *meso*-tetra-(4-sulfonatophenyl)-tetrabenzoporphyrin was purchased from Porphyrin Products (Logan, UT). Glucose oxidase (powder from *Aspergillus niger),* D (+) glucose anhydrous, Histopaque-1077 and remaining reagents were purchased from Sigma-Aldrich (St. Louis, MO).

Pd phosphor solution (2.5 mg/ml = 2 mM) was prepared in distilled water (dH_2_O) and stored at −20°C. Glucose oxidase solution was prepared in dH_2_O (10 mg/mL) and stored at −20°C. Sodium cyanide (NaCN) solution (1.0 M) was prepared in dH_2_O; the *p*H was adjusted to ~7.0 with 12N HCl and stored at −20°C. Phosphate-buffered saline (PBS) containing glucose (137 mM NaCl, 2.7 mM KCl, 4.3 mM Na_2_HPO_4_, 1.4 mM KH_2_PO_4_ and 10 mM glucose; *p*H 7.4) was stored at 4°C.

### Subjects

Venous blood samples (5 to 8 mL) were collected in heparin tubes and processed in <2 hr for peripheral blood mononuclear cell (PBMC) isolation and O_2_ measurement. Blood was also collected from age- and gender-matched healthy controls. All trisomy 21 participants attended the outpatient facilities at Tawam and Al Ain Hospitals (Al Ain city, Abu Dhabi) for routine visits. All control participants were healthy children who had no medical complaints.

The study was approved by the institutional review board for protection of human subjects. Informed consent was obtained for each participating subject.

### PBMC isolation

Plasma was collected from blood samples by centrifugation and possessed for Comprehensive Metabolic Panel and lipid profile. The samples were then diluted with equal volume of phosphate-buffered saline (PBS) containing 10 mM glucose and gently layered on the top of 10 mL Histopaque-1077. The mixtures were centrifuged at 15°C, 400 x*g* for 30 min. Collected PBMC were diluted with the same solution and re-centrifuged as above. The pellets were suspended in PBS, 10 mM glucose, 3 μM Pd phosphor and 0.5% fat-free bovine serum albumin for O_2_ measurements at 37°C. Cell count and viability were determined by light microscopy, using a hemocytometer under standard trypan blue staining conditions. Only trypan blue-negative cells (>95%) were counted.

### Oxygen instrument

A phosphorescence oxygen analyzer that measures dissolved O_2_ in solutions as function of time was used to determine the rate of PBMC respiration [12,13]. This method is based on the principle that O_2_ quenches the phosphorescence of a palladium phosphor [14].

The Pd (II) derivative of *meso*-tetra-(4-sulfonatophenyl)-tetrabenzoporphyrin had an absorption maximum at 625 nm and a phosphorescence emission maximum at 800 nm. Samples were exposed to light flashes (10 per sec) from a pulsed light-emitting diode array with a peak output at 625 nm. Emitted phosphorescent light was detected by a Hamamatsu photomultiplier tube after first passing it through a wide-band interference filter centered at 800 nm. Amplified phosphorescence was digitized at 1–2 MHz using an analog/digital converter (PCI-DAS 4020/12 I/O Board) with 1 to 20 MHz outputs.

The phosphorescence decay rate (1/τ) was characterized by a single exponential; I = Ae^-*t*/τ^, where I = Pd phosphor phosphorescence intensity. The values of 1/τ were linear with dissolved O_2_: 1/τ = 1/τ^o^ + *k*_*q*_[O_2_, where 1/τ = the phosphorescence decay rate in the presence of O_2_, 1/τ^o^ = the phosphorescence decay rate in the absence of O_2_, and *k*_q_ = the second-order O_2_ quenching rate constant in sec^-1^ μM^-1^ (14). For calibration, the reaction contained PBS, 3 μM Pd phosphor, 0.5% fat-free albumin, 50 μg/mL glucose oxidase and various concentrations of β-glucose [11].

Cellular respiration was measured at 37°C in 1.0-mL sealed vials. Mixing was carried out with the aid of parylene-coated stirring bars. The respiratory substrates were endogenous metabolic fuels supplemented with glucose.

### Statistical analysis

The data are summarized by arithmetic mean and standard deviation. Mann–Whitney *U* test was used for nonparametric values. *P*<0.05 was considered significant.

## Results

In cell suspensions sealed from air, [O_2_] decreased linearly with time, indicating the kinetics of mitochondrial O_2_ consumption was zero-order. The rate of respiration (*k*, in μM O_2_/min) was thus the negative of the slope d[O_2_]/d*t*. Cyanide markedly inhibited respiration (≥96%), confirming O_2_ was consumed mainly by the mitochondrial respiratory chain.

**Figure 1:**
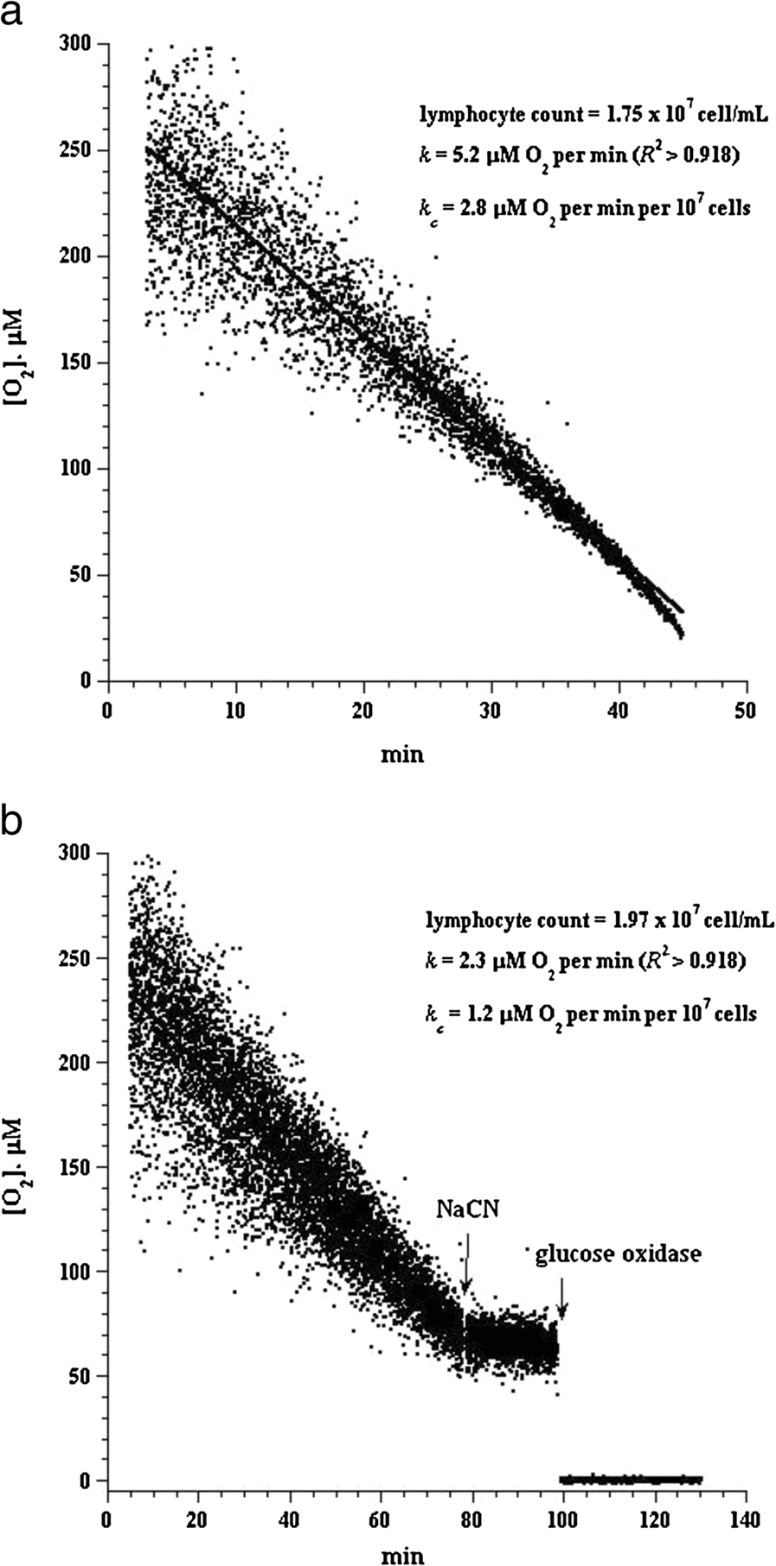
**Representative O**_**2**_**runs for lymphocyte respiration in a 15-year-old male with trisomy 21 (Panel a, Subject 8 in Table**1**) and control subject (Panel b).** The lines are best linear fits (*R*^2^ >0.830). The additions of 10 mM NaCN and 50 μg/mL glucose oxidase are shown.

**Figure 2:**
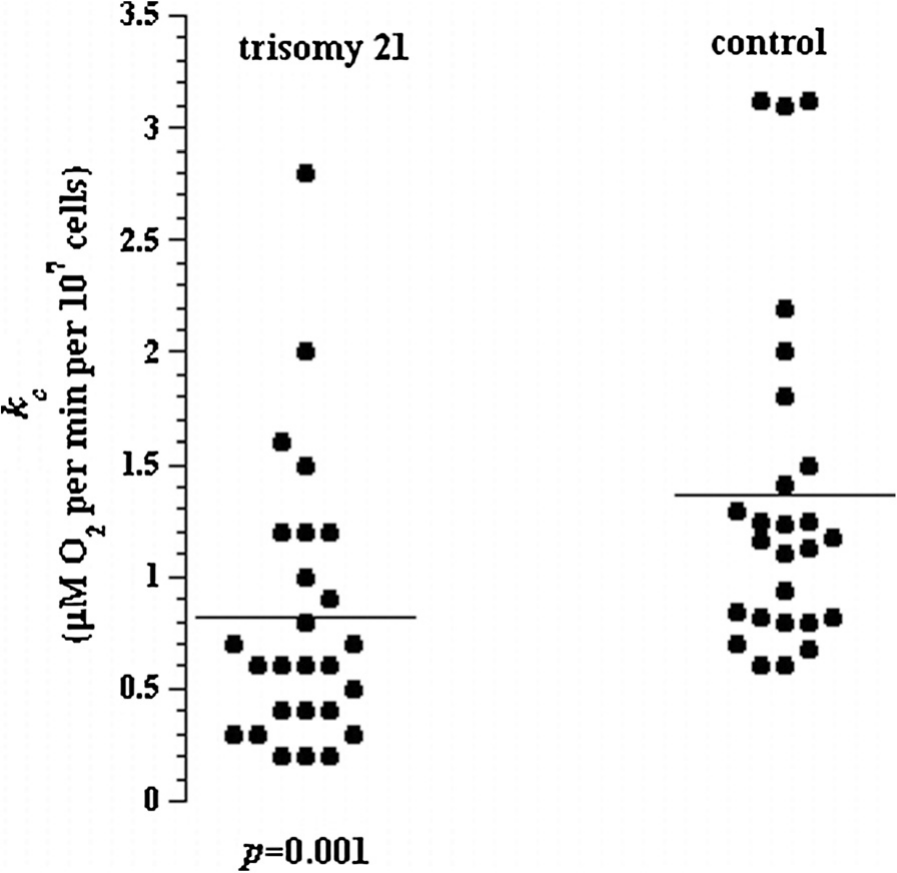
**Lymphocyte respiration in 26 children with trisomy 21 and 26 control children.** The horizontal lines are mean values.

Lymphocyte respiration was measured in 26 children with trisomy 21 and 26 control children. Representative O_2_ runs are shown in Figure 1a-b. For trisomy 21 children, the rate of respiration (*k*_*c*_, in μM O_2_ per min per 10^7^ cells, mean ± SD, n = 26) was 0.82 ± 0.62 (median = 0.60; range = 0.20 to 2.80), Table 1. The values of *k*_*c*_ for control children (n = 26) were 1.36 ± 0.79 (median = 1.17; range = 0.60 to 3.12; -2SD = 0.61). The *p* value for *k*_*c*_ between trisomy 21 and control children was <0.001, Figure 2. Similar results with higher significance (*p*<0.000) were obtained after excluding the five children with trisomy 21 and elevated serum TSH (values >6.1 mU/L). Fourteen of 26 (54%) children with trisomy 21 had *k*_*c*_ values of 0.20 to 0.60 (<−2SD).

**Table 1:** Rates of lymphocyte respiration, thyroid function and clinical findings in trisomy 21 children

**Children No.**	**Age (yr)**	**Gender**	**BMI (kg/m**^**2**^**)**	***k***_***c***_**(μM O**_**2**_**min**^**-1**^**per 10**^**7**^**cells)**	**TSH (mIU/L)**	**Free T4 (pmol/L)**	**Clinical status**
1	1.5	F	12.8	1.6	3.5	20.7	AVSD
2	5	M	16.1	2.0	1.6	14.7	celiac disease
3	17	M	28.6	1.2	4.9	9.5	normal
4	12	M	16.3	0.6	2.6	10	AVSD
5	12	F	30.8	1.2	3.8	13.2	VSD
6	9	M	17.1	0.6	2.6	NA	bronchial asthma
7^*^	15	M	30.2	0.6	2.8	10.0	aortic stenosis, hypothyroidism
8	15	M	44.3	2.8	13.2	11.0	Moya Moya disease, hypothyroidism
9	3	F	13.8	0.4	3.7	14.2	bronchial asthma
10	2	F	15.2	0.6	5.3	13.0	obstructive sleep apnea, low vitamin D
11	3	M	14.5	0.5	2.9	15.8	ASD, PDA
12	5	M	17.5	0.9	2.6	9.9	VSD
13^*^	4	M	15.7	0.2	12.6	11.6	hypothyroidism
14	10	F	NA	0.8	5.1	10.4	normal
15	6	M	18.7	0.4	5.1	10.5	normal
16	4	M	14.8	1.2	3.6	11.9	normal
17	9	F	16	0.7	3.9	10.7	normal
18	1.5	M	16.3	1.5	11.6	11.9	hypothyroidism
19	5	M	NA	1.0	2.9	11.1	normal
20	6	M	20.7	0.3	3.9	10.4	normal
21	8	M	31.4	0.4	5.1	10.5	asthma, myelomeningocele
22	2	F	14.3	0.3	7.7	11.2	normal
23	7	F	17.8	0.3	1.3	9.3	normal
24	8	F	23.2	0.2	3.7	11.3	AVSD
25	6	M	35.1	0.2	3.6	14.1	AVSD
26	4	M	13.6	0.7	6.4	14.1	normal
mean ± SD (CV)	6.9 ± 4.4		20.6 ± 8.3 (40%)	0.82 ± 0.62 (76%)	4.8 ± 3.1	12.0 ± 2.5	

AVSD, atrioventricular septal defect; VSD, ventricular septal defect; ASD, atrial septal defect; PDA, patent ductus arteriosus; CV, coefficient of variation. NA, not available.

In normal 2 to7 year-old children, the TSH values are 0.10 to 5.9 mU/L (mean = 2.2 mU/L); in normal 9 to 16 year-old children, the TSH values are 0.20 to 6.1 mU/L (mean = 2.3 mU/L).

Reference ranges for free T4 are 11.0 to 22.6 pmol/L in children 1 to 5 years of age, 11.6 to 21.5 pmol/L in children 6 to 10 years of age, and 12.0 to 20.6 pmol/L in children 11 to 19 years of age ( https://www.labcorp.com/wps/portal/provider).

* On thyroxin prior to O_2_ consumption testing.

**Figure 3:**
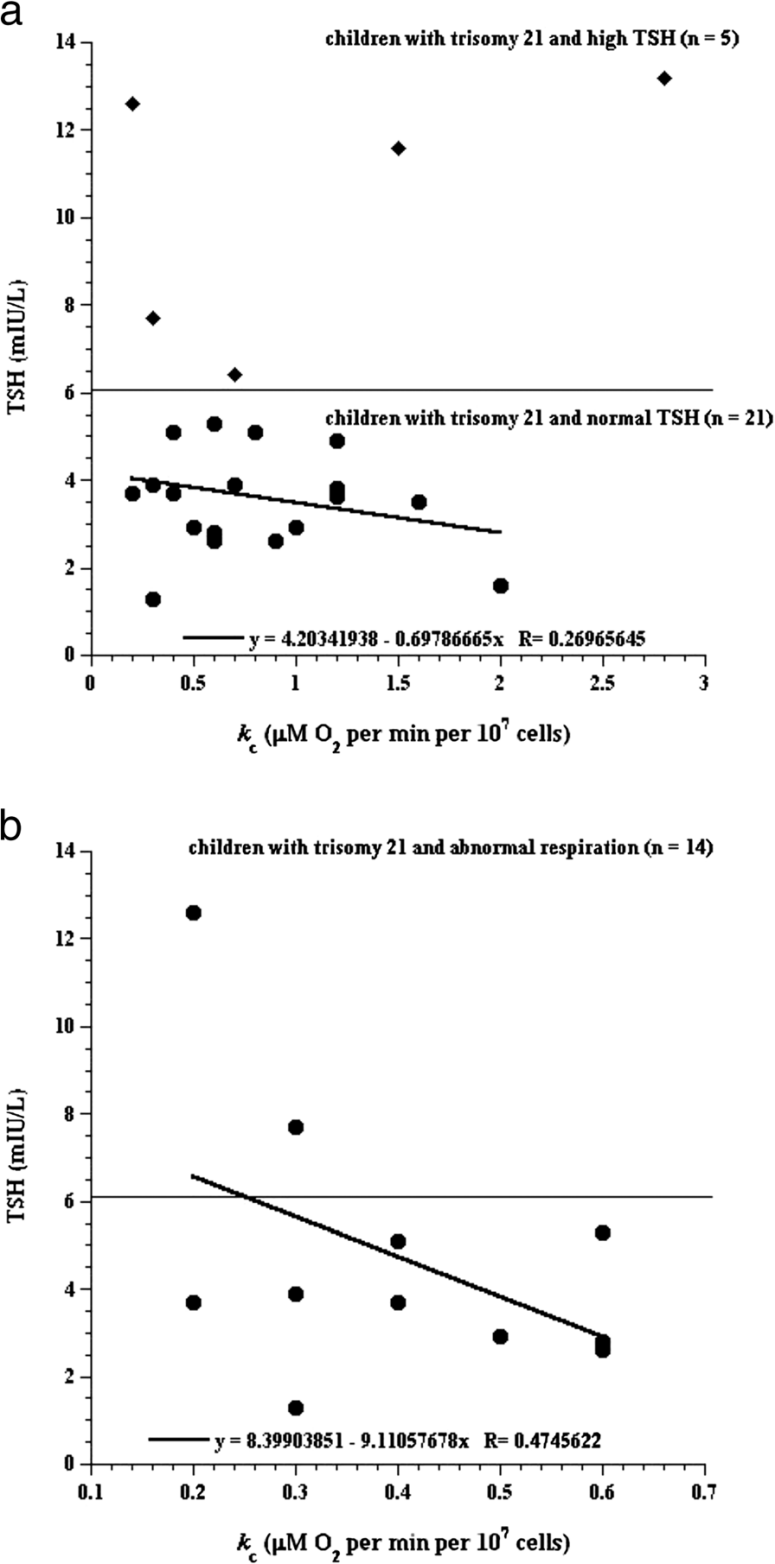
**Lymphocyte respiration in children with trisomy 21 as a function of serum TSH. *Panel a:*** Circles, children (n = 21) with trisomy 21 and normal TSH (levels ≤5.3 mU/L; line is the best linear fit, *R*^2^ > 0.0727); diamonds, children (n = 5) with trisomy 21 and elevated TSH (levels 7.7 to 13.2 mU/L). ***Panel b:*** Children (n = 14) with trisomy 21 and abnormal (low) rate of respiration (*k*_*c*_ < 0.60 μM O_2_ per min per 10^7^ cells). The horizontal line reflects upper limit of normal TSH (<6.1 mU/L, please see footnote to Table 1).

In children with trisomy 21 and normal TSH (n = 21), the *k*_*c*_ value did not correlate with the TSH level (*R*^*2*^ >0.072, Figure 3a). By contrast, in children with trisomy 21 and abnormal lymphocyte respiration (*k*_*c*_ < 0.61, n = 14), the *k*_*c*_ value correlated with the TSH level (*R*^*2*^ >0.225, *R* >0.474, Figure 3b).

Five children with trisomy 21 had elevated TSH levels (>6.1 mIU/L). Their median TSH was 12.6 mIU/L (range, 6.4 to 13.2) and median *k*_*c*_ was 0.7 μM O_2_ per min per 10^7^ cells (range, 0.2 to 2.8). Subject 8 (15-year-old adolescent male) had a TSH level of 13.2 mIU/L and a *k*_*c*_ values of 2.8 μM O_2_ per min per 10^7^ cells (Table 1 and Figure 1a).

There were 8 children with trisomy 21 and congenital heart disease. Their median *k*_*c*_ value was 0.6 μM O_2_ per min per 10^7^ cells (range, 0.2 to 1.6), and did not significantly differ from the remaining children (*p* = 0.238).

**Figure 4:**
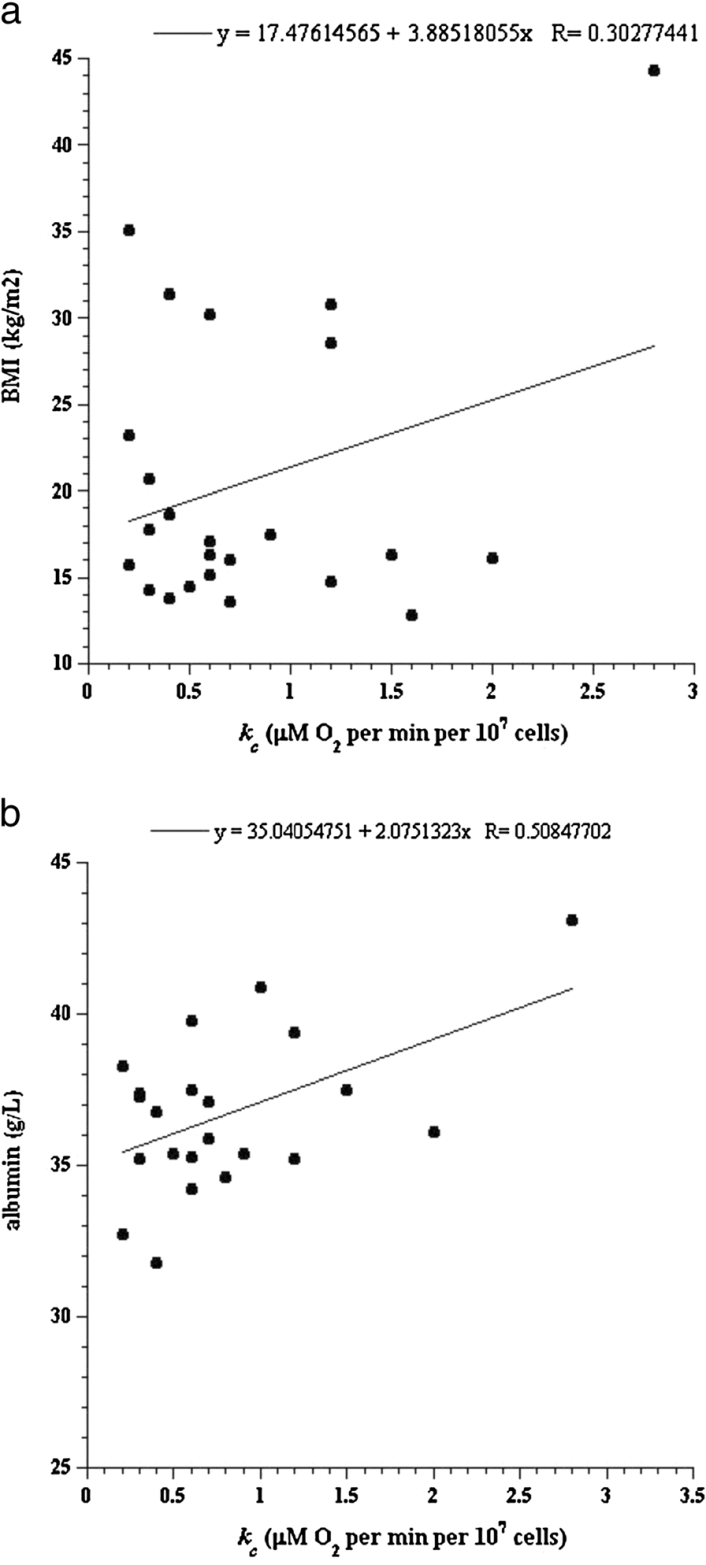
**BMI (Panel a) and serum albumin (Panel b) as a function of rates of lymphocyte respiration (*k***_***c***_**) in trisomy 21 children.**

In children with trisomy 21, the *k*_*c*_ positively correlated with BMI (*R* >0.302, Figure 4a), serum creatinine (*R* >0.507), BUN (*R* >0.535) and albumin (*R* >0.446, Figure 4b), Table 2.

**Table 2:** Correlations (*R*) between serum metabolic parameters and rates of lymphocyte respiration in trisomy 21 (n = 23) and control (n = 25) children^*^

											**Rate of respiration (*kc*)**											
	**Creatinine**	**BUN**	**Total protein**	**Albumin**	**Ca**^**2+**^	**Glucose**	**Osmolality**	**Cholesterol**	**HDL**	**LDL**	**TG**	
Trisomy 21	0.507	0.535	0.446	0.508	0.137	0.048	0.007	0.091	0.145	0.092	0.170
Control	0.192	0.037	−0.146	−0.091	−0.136	0.112	−0.009	−0.060	−0.080	−0.024	−0.057

* Serum was not available in 3 patients and 1 control.

## Discussion

The rates of lymphocyte respiration in the children with trisomy 21 were slower than in the control group (Figure 2). These differences could reflect a relatively lower rate of mitochondrial energy conversion in trisomy 21 children that may be linked to some pathological findings pertinent to this disorder, such as defects in the inner mitochondrial membrane [5,8].

The mechanism for slower rates of lymphocyte respiration in children with trisomy 21 could be multi-factorial. For example, the thyroid hormone is a well known regulator of the rate of metabolism; and hypothyroidism is common in children with trisomy 21. As shown in Figure 3b, high TSH (low or ineffective thyroxin) may contribute to the slower lymphocyte respiration in some children. Thus, thyroxin replacement is expected to improve lymphocyte respiration in those with hypothyroidism.

Of note, normal TSH values for children 2 to 7 years of age are 0.10 to 5.9 mU/L (mean = 2.2 mU/L) and for children 9 to 16 years of age are 0.20 to 6.1 mU/L (mean = 2.3 mU/L). Using these cutoffs, lymphocyte respiration was found to be higher in euthyroid trisomy 21 children than those with hypothyroidism.

Body-mass index, protein metabolism (BUN, total protein and albumin), and serum creatinine positively correlated with rates of lymphocyte respiration, but only in trisomy 21 children (Table 2 and Figure 4a-b). As previously reported, protein metabolism (proteolysis, oxidation and synthesis) is linked to obesity [15,16], a finding that is common in children with trisomy 21.

Close correlations were documented between cerebral O_2_ consumptions and mental function, including depression and dementia [17,18]. It is unknown if our finding of slower lymphocyte respiration in trisomy 21 children is applicable to other organs. Nevertheless, our findings are consistent with the recent reports on mitochondrial disturbances in those with trisomy 21 [19-23]. Decreased basal 3'-5'-cyclic adenosine monophosphate, increased reactive oxygen species and impaired NADH:ubiquinone reductase (complex I of the respiratory chain) were noted in fibroblasts from those with trisomy 21 [20].

### Limitations of the study

No study was found in the literature that addressed lymphocyte respiration in children with trisomy 21. Additional studies are needed in a larger population.

## Conclusions

Children with trisomy 21 in this study have lower lymphocyte bioenergetics, a finding that is consistent with the known mitochondrial disturbances in these children. The clinical significance implication of this finding requires further studies.

## Competing interests

The authors declare that they have no competing interests.

## Authors’ contributions

EHA and AKS designed the study, carried out the analysis, interpreted the data and wrote the manuscript. Both authors read and approved the final manuscript.

## Pre-publication history

The pre-publication history for this paper can be accessed here:

http://www.biomedcentral.com/1471-2431/12/193/prepub
